# Novel insights into antimicrobial-resistant, virulent and biofilm-forming *Salmonella*: Molecular and phenotypic evidence from duck at the human-animal-environment interface

**DOI:** 10.3389/fmicb.2025.1753559

**Published:** 2026-01-23

**Authors:** Aditya Paul, Siddhartha Narayan Joardar, Indranil Samanta, Kunal Batabyal, Samir Dey, Prakash Ghosh, Ahmed Abd El Wahed, Rajarshi Bardhan, Keshab Chandra Dhara, Sanjoy Datta

**Affiliations:** 1 Department of Veterinary Microbiology, Faculty of Veterinary and Animal Sciences, West Bengal University of Animal and Fishery Sciences, Kolkata, India; 2 Institute of Animal Hygiene and Veterinary Public Health, University of Leipzig, Leipzig, Germany; 3 Directorate of Research, Extension and Farms, West Bengal University of Animal and Fishery Sciences, Kolkata, India; 4 Department of Animal Genetics & Breeding, Faculty of Veterinary and Animal Sciences, West Bengal University of Animal and Fishery Sciences, Kolkata, India

**Keywords:** antibiotic resistance, biofilm, duck, phylogenetic tree, *Salmonella* spp., virulence, Western blot

## Abstract

The present study provides first time the comprehensive molecular and phenotypic characterization of antimicrobial-resistant, biofilm-forming, and virulent *Salmonella* spp. isolated from apparently healthy ducks and their environments in West Bengal, India. A total of 462 samples from Indigenous, Khaki Campbell, and Pekin ducks yielded 436 isolates, of which 42.2% were ESBL producers carrying *bla*_*TEM*_ (36.5%), *bla*_*CTX*−*M*_(20.6%), *bla*_*SHV*_ (17.7%), and *bla*_*AmpC*_ (32.6%). Sequence analysis revealed multiple clinically relevant alleles, including *bla*_*TEM*_-164, *bla*_*CTX*−*M*_-15, and *bla*_*SHV*_-45, underscoring their potential public health significance. The isolates were also screened for biofilm genes (*csgA, sdiA, rpoS, rcsA*), and the virulence gene *invA*. Biofilm-associated genes were widely distributed (*csgA*: 54.59%, *sdiA*: 52.52%, *rpoS*: 80.28%, *rcsA*: 63.76%), while 141 (32.34%) of isolates possessed the *invA* virulence marker. Of 26 selected strains, high multi-drug resistance was detected, mainly against tetracycline and cefixime. Phylogenetic analysis of ESBL gene sequences showed clustering across avian, animal, and clinical (human) *Salmonella* isolates, indicating potential interspecies transmission and evolutionary divergence. Notably, strong positive correlations were observed among biofilm formation, multidrug resistance, and virulence (τ = 0.656, ρ = 0.765, *p* < 0.001). Western blotting further identified two unique polypeptide markers (69 and 35 kDa) with diagnostic potential for detecting resistant, virulent, and biofilm-forming *Salmonella*. In short, these findings highlight, for the first time, duck as silent reservoirs of high-risk *Salmonella* strains, and propose novel protein markers to facilitate early detection at the human-animal-environment interface.

## 1 Introduction

*Salmonella*, one of the prominent Gram negative bacteria, stands as a paramount concern in the realm of public health due to its profound pathogenic potential. *Salmonella* causes great losses in chicken and duck (Shehata et al., 2019) and are found responsible for a spectrum of infections ranging from gastroenteritis to severe systemic diseases, the resilience and adaptability of *Salmonella* present ongoing challenges in both clinical and environmental settings. In recent years, the increasing emergence and spread of multidrug-resistant (MDR) *Salmonella*, in humans and animals has been reported worldwide (Frye and Fedorka-Cray, 2007; Eller et al., 2013; Djeffal et al., 2017). High concentration of unsaturated fatty acids and organoleptic properties increased the consumer demand of duck meat in recent times, although, the research gaps exists for ducks as a source of food borne pathogens (An et al., 2025). One of the intriguing aspects of this bacterium lies in its genetic repertoire, encompassing genes that govern biofilm formation, antibiotic resistance, and virulence and single-sought identification scheme of the detrimental triad is a crucial research gap. Moreover, *Salmonella* has been reported as one of the most common causes of food poisoning in humans (Kim and Lee, 2017). It was estimated that over 93.8 million of *Salmonella* gastroenteritis, with 155,000 deaths, occur globally each year (Majowicz et al., 2010). *Salmonella* infections in humans have commonly been associated with the consumption of contaminated animal origin foods, especially poultry meat and eggs (Im et al., 2015; Eguale et al., 2017; Delahoy et al., 2018; McWhorter and Chousalkar, 2019). Biofilm formation, facilitated by few proteins encoded by *csgA, rcsA, sdiA*, and *rpoS* genes, allows *Salmonella* to adhere to surfaces and form structured communities, enhancing its survival in diverse environments and increasing resistance to disinfectants and antibiotics. Concurrently, the emergence and dissemination of antibiotic resistance genes, particularly extended-spectrum beta-lactamase (ESBL) genes (*bla*_*TEMType*_, *bla*_*SHVType*_, *bla*_*CTX*−*MType*_) and AmpC beta-lactamase (ACBL) gene (*bla*_*AmpC*_), pose significant therapeutic challenges, limiting treatment options and complicating infection control measures. Furthermore, the virulence factor *invA*, a linchpin in *Salmonella* pathogenesis, orchestrates the bacterium's adeptness in invading host cells and inciting deleterious infections. The *inv*A gene of *Salmonella* contains sequences unique to this genus and has been proved as a suitable PCR target with potential diagnostic application (Rahn et al., 1992). This study undertakes a comprehensive approach to characterize biofilm-forming and antibiotic resistant *Salmonella* isolates from ducks and their environment by integrating molecular as well as phenotypic analyses. The study further incorporates nucleotide sequencing to explore the genetic diversity of resistance genes and employs antibiotic susceptibility testing (ABST) to evaluate resistance profiles. Additionally, the diagnostic potential of isolated proteins as markers was assessed for detection of *Salmonella* with antimicrobial resistance, biofilm formation and virulence genes.

## 2 Materials and methods

### 2.1 Sample

**Figure 1:**
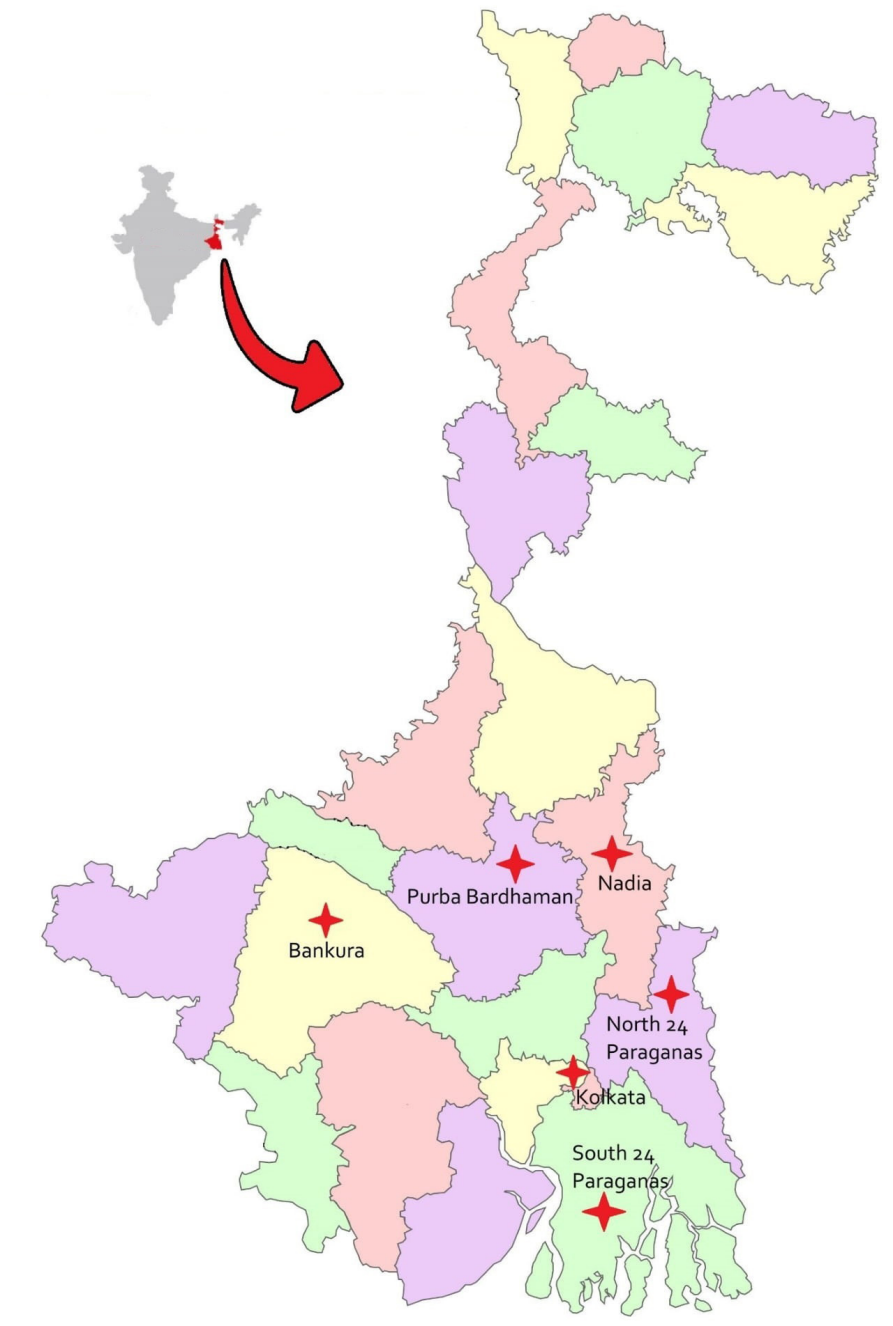
Sites of sample collection in West Bengal state.

A total of 462 samples were collected including 215 tracheal swabs, 208 cloacal swabs, and 39 environmental samples from Indigenous (non-descriptive), Khaki Campbell, and Pekin ducks, along with their associated environments. The environmental samples consisted of soil (*n* = 13), water from duck environments/sheds (*n* = 13), and duck feed (*n* = 13). Sampling was conducted randomly from apparently healthy ducks reared under semi-intensive system across various southern districts of West Bengal (22.98° N, 87.85° E**)**, India (Figure 1). The ducks were maintained by home-made/natural (environmental) feed without antibiotic exposure (therapeutic and non-therapeutic/growth promoter). The study included eight duck farms, typically characterized by small flock sizes maintained under backyard or free-range conditions, which are common in rural and peri-urban areas and often support supplementary livelihoods.

### 2.2 Sample collection and processing

Tracheal and cloacal samples were collected aseptically using sterile cotton swabs (Hi-Media, India) following the guidelines recommended by the Office International des Epizooties (OIE, 2008). Immediately after collection, swabs were placed in sterile peptone water (HiMedia, India), which was used solely as a transport medium to maintain sample integrity during transfer from the field to the laboratory. Feed, drinking water from duck environment/sheds, and soil samples were collected aseptically using a sterile spatula and transferred into sterile collection vials (Hi-Media, India). All samples, including swabs suspended in peptone water, were properly labeled, kept on ice in a thermo flask and transported to the laboratory for further processing. Upon arrival at the laboratory, cloacal and tracheal swabs were removed from the peptone water and subjected to selective enrichment in Selenite F broth (HiMedia, India). Feed and soil samples were inoculated directly into for further processing. Upon arrival at the laboratory, cloacal and tracheal swabs were removed from the peptone water and subjected to selective enrichment in Selenite F broth (HiMedia, India) Feed and soil samples were directly inoculated into the Selenite F broth (4 g of sample into 36 mL of broth), while the drinking water samples were inoculated at a ratio of 5 mL sample to 45 mL Selenite F broth (Samanta et al., 2014). All enrichment cultures were then incubated at 37 °C for 24 hours in an incubator (Digitech Systems, India) to facilitate selective recovery of *Salmonella*.

### 2.3 Isolation and identification of bacteria

To isolate *Salmonella* spp., swabs from peptone water were aseptically inoculated into Selenite F broth (Hi-Media, India) and incubated at 37 °C for 24 h. Cultures with turbidity and often by a color change of the medium to pink-orange-red were selected, streaked into Xylose Lysine Deoxycholate (XLD) Agar (Hi-Media, India), and incubated overnight at 37 °C. Colonies with red color and black centers were identified and sub-cultured in sterile Nutrient agar slants for subsequent morphological and biochemical characterization. *Salmonella* isolates were confirmed through standard biochemical tests, viz. Catalase, Oxidase, Indole, Methyl-red, Voges-Proskauer, Citrate, and Urease tests (Quinn et al., 1994).

### 2.4 DNA Extraction from *Salmonella* isolates

For molecular characterization, *Salmonella* spp. isolates preserved in Nutrient agar slants were transferred into sterile nuclease-free water (Promega, USA) in 1.5 ml centrifuge tubes (Tarsons, India). Bacterial cells were lysed by boiling in a water bath for 10–15 min, followed by immediate chilling. Cell debris was removed by centrifugation (Hermle Z216-MK Refrigerated Microcentrifuge, Germany) at 4 °C, 1,900 g for 5 min. The resulting supernatants served as template DNA for PCR reactions (Wani et al., 2004).

### 2.5 Molecular characterization of *Salmonella* isolates

Morphologically and biochemically confirmed *Salmonella* spp. isolates were subjected to PCR for molecular confirmation using the primers targeting the *Salmonella*-specific 16S rRNA gene (Pradhap et al., 2011). PCR amplification was carried out using standard reagents (Promega, USA) and primers (IDT, USA) in a gradient thermocycler (BIO-RAD T100, USA). Each reaction was performed in a total volume of 25 μL containing 5μl of extracted genomic DNA 5μl of 5X Green GoTaq Flexi Buffer, 1μl of MgCl_2_ (25mM), 0.5μl of dNTP mix (10mM), 0.2μl of each primer (100μM), and 0.25μlof Taq DNA polymerase (5U/μl). The primer sequences, annealing temperatures, and expected amplicon sizes are summarized in Table 1.

**Table 1:** PCR primer sequences, annealing temperatures, and product sizes used for the PCR in this study.

**Gene**	**Primer**	**Oligonucleotide sequence (5^′^ → 3^′^)**	**Annealing temperature**	**Product size (bp)**	**Reference**
*Salmonella* 16S rRNA	Forward	AGAGTTTGATCMTGGCTCAG	50 °C	1,428	Pradhap et al., 2011
	Reverse	TACGGYTACCTTGTTACGACTT			
*bla_*TEM*_*	Forward	ATAAAATTCTTGAAGACGAAA	53 °C	1,080	Weill et al., 2004
	Reverse	GACAGTTACCAATGCTTAATC			
*bla_*SHV*_*	Forward	TTATCTCCCTGTTAGCCACC	54.4 °C	795	Cao et al., 2002
	Reverse	GATTTGCTGATTTCGCTCGG			
*bla_*CTX*−*M*_*	Forward	CAATGTGCAGCACCAAGTAA	53 °C	540	Weill et al., 2004
	Reverse	CGCGATATATCGTTGGTGGTTGGTG			
*bla_*AmpC*_*	Forward	CCCCGCTTATAGAGCAACAA	58.1 °C	634	Féria et al., 2002
	Reverse	TCAATGGTCGACTTCACACC			
*csgA*	Forward	ATCTGACCCAACGTGGCTTCG	55 °C	178	Silva et al., 2013
	Reverse	GATGAGCGGTCGCGTTGTTACC			
*rpoS*	Forward	GCAGAGCATCGTCAAATGGCTGTT	60 °C	120	Adamus-Białek et al., 2015
	Reverse	ATCTTCCAGTGTTGCCGCTTCGTA			
*rcsA*	Forward	GTGATTCACAGCGCCCTTCA	54 °C	306	Adamus-Białek et al., 2015
	Reverse	TACTCGATTCGGTTCGGCTC			
*sdiA*	Forward	TCGCTATCTCTGCTGATGTC	52 °C	239	Adamus-Białek et al., 2015
	Reverse	TTTAATGCTGCCAAATCGGG			
*invA*	Forward	GTGAAATTATCGCCACGTTCGGGCAA	64 °C	1,428	Salehi et al., 2005
	Reverse	TCATCGCACCGTCAAAGGAACC			

### 2.6 Phenotypic confirmatory test for production of ESBL in *Salmonella* spp. isolates

Antibiotic disc containing cefotaxime (30μg, Hi-Media, India) and ceftazidime disks (30 μg, Hi-Media, India) with and without clavulanate (10μg, Hi-Media, India) were used in disc diffusion method for phenotypic confirmation of the presence of ESBLs in *Salmonella* spp. isolates. A difference of ≥ 5mm between the zone of inhibition in presence and absence of clavulanate was considered for phenotypic confirmation of ESBL production.

### 2.7 Detection of ESBL and ACBL genes in *Salmonella* isolates

All the positive *Salmonella* isolates were subjected to PCR for detection of major antibiotic-resistant genes, viz. *bla*_*TEM*_, *bla*_*SHV*_, *bla*_*CTX*−*M*_ and *bla*_*AmpC*_(Weill et al., 2004; Cao et al., 2002; Féria et al., 2002) using gene specific primers In brief, 5μl of DNA templates, 5 μl of 5X Green GoTaq Flexi Buffer, 2 μl MgCl_2_ (25 mM), 2.5 μl of Dimethyl Sulphoxide (SRL, India), 0.5 μl of dNTP (10mM), lμl of each primer (100 μM) [0.5 μl for *bla*_*CTX*−*M*_and 0.2 μl for *bla*_*SHV*_] and 0.25 μl of Taq DNA polymerase (5U/μl) were added. The primer sequences, annealing temperatures, and expected amplicon sizes for each gene are provided in Table 1.

The commercial source (Barcode Bioscience, Bangalore, Indiaand Nalgen Bio Private Limited, West Bengal, India) was used for the sequencing of selected PCR products [8 PCR products of *bla*_*SHV*_ gene, 5 PCR products of *bla*_*CTX*−*M*_ gene and 4 PCR products of *bla*_*TEM*_ gene]. The sequence homology searches were conducted using the BLAST algorithm (http://blast.ncbi.nlm.nih.gov/Blast.cgi). The sequence homology was detected by the standard nucleotide BLAST algorithm (https://blast.ncbi.nlm.nih.gov/Blast.cgi?CMD=Web&PAGE_TYPE=BlastHome). The sequences were then submitted to the DNA Data Bank of Japan (DDBJ; www.ddbj.nig.ac.jp).

### 2.8 Determination of biofilm forming *Salmonella* spp. isolates by tissue culture plate method

The quantitative test, considered as gold-standard for biofilm detection (Christensen et al., 1985), was used in this study. In brief, organisms isolated from fresh agar plates were inoculated in 10 mL of trypticase soy broth with 1% glucose. Broths were incubated at 37 °C for 24 h and the cultures were diluted 1:100 with fresh medium. Individual wells of sterile 96 well-flat bottom polystyrene tissue culture plates (Sigma-Aldrich, Costar, USA) were filled with 200 μl of the diluted cultures. The plates were incubated at 37 °C for 24 h and the contents of each well were removed by gentle tapping. The wells were washed with 0.2 mL of phosphate buffer saline (pH 7.2) four times. Biofilm formed by bacteria adherent to the wells were fixed by 2% sodium acetate and stained by crystal violet (0.1%). Optical density (OD) of stained adherent biofilm was obtained by using micro ELISA reader (model Merilyzer EIAQUANT, Meril, India) at wavelength 570 nm. The interpretation of biofilm production was done (StepanoviC et al., 2007) which is as follows:

**Table:** 

**Average OD Value**	**Biofilm Production**
O.D._isolate_ ≤ O.D._C_	Non-biofilm producer
O.D._C_ ≤ O.D._isolate_ ≤ 2O.D._C_	Weak biofilm producer
2O.D._C_ ≤ O.D._isolate_ ≤ 4O.D._C_	Moderate biofilm producer
4O.D._C_ ≤ O.D._isolate_	Strong biofilm producer

### 2.9 Detection of biofilm genes in *Salmonella* isolates

All positive *Salmonella* isolates underwent PCR to detect biofilm genes, including *csgA, rpoS, rcsA*, and *sdiA* (Silva et al., 2013; Adamus-Białek et al., 2015) with specific primers. PCR assays were performed in a final reaction volume of 25 μL containing 5 μl DNA templates, 5 μl of 5X Green GoTaq Flexi buffer, 2 μl MgCl_2_ (25 mM), 0.5 μl of dNTP mix (10 mM), 0.1 μl of each gene-specific primers (100 μM) and 0.5 μl Taq DNA polymerase (5U/ μl) (GCC Biotech, India). The primer sequences, annealing temperatures, and expected amplicon sizes for each target gene are summarized in Table 1.

### 2.10 Detection of Virulence gene in *Salmonella* isolates

All confirmed *Salmonella* isolates were subjected to PCR to detect the virulence-associated *invA* gene using gene-specific primers (Salehi et al., 2005) PCR amplification was carried out in a final reaction volume of 25 μL containing 5 μl of the template DNA 5 μl of 5 X Green GoTaq Flexi buffer, 2 μl MgCl_2_ (25 mM), 0.5 μl of dNTP mix (10mM), 0.2 μl of each gene-specific primers (100 μM) and 0.25 μl of Taq DNA polymerase (5 U/μl) (Promega, USA). The primer sequences, annealing temperatures, and expected amplicon sizes are summarized in Table 1.

### 2.11 Electrophoresis of PCR products

PCR amplicons were analyzed by agarose gel electrophoresis using 1.5% agarose (SRL, India) prepared in 1X TAE buffer and stained with ethidium bromide (0.5 μg/mL; SRL, India). Electrophoresis was carried out at a constant voltage of 120 V, and the amplified products were visualized under UV illumination (Labnet Enduro^R^, GDS-1302, USA). A 100 bp DNA ladder (SRL, India) was used as a molecular size marker for amplicon size determination.

### 2.12 *In vitro* Antibiotic Sensitivity test (ABST) of ESBL, biofilm producing and virulence gene carrying *Salmonella* isolates

All the *Salmonella* isolates (*n* = 26) carrying ESBL, biofilm producing and virulence gene were tested for their sensitivity/resistance to 10 different antibiotics by the disc diffusion method (Clinical Laboratory Standards Institute, 2020). The antibiotics used were Amoxicillin (AMX 10 μg), Cefixime (CFM 10 μg), Chloramphenicol (C 25 μg), Co-Trimoxazole (Cm 25 μg), Doxycycline (DO 30 μg), Enrofloxacin (EX 5 μg), Gentamicin (Gen 10 μg), Imipenem EDTA (IE 10/75U) and Tetracycline (TE 30 μg), Ticarcillin/Clavulanic acid (TCC 75/10 μg).

### 2.13 Assessing partial clonal relationship of duck origin β-Lactamase producing *Salmonella* strains with human clinical isolates and diverse animal hosts

The selected β-lactamase sequences from the present study were compared with the ESBL sequences of clinical *Enterobacteriaceae* strains isolated from human patients and those from poultry, cattle, dog, cat, bats, pigs, and giant panda in India and other subcontinents (Australia, Algeria, Brazil, China, Bangladesh, China, Italy, Japan, Myanmar, Saudi Arabia, South Korea, Thailand), available in the NCBI-Genbank database (National Centre for Biotechnology Information; https://www.ncbi.nlm.nih.gov/genbank/). The phylogenetic tree was constructed by the maximum likelihood (ML) method using molecular evolutionary genetics analysis (MEGA-X; https://www.megasoftware.net/) and analyzed in iTOL v7 (https://itol.embl.de/).

### 2.14 Association of antibiotic resistance, biofilm and virulence gene prevalence

The biofilm formation and antibiotic resistance gene expressions of the samples were taken as ordinal variables (*i.e*. absent, mild, moderate, strong and very strong) and virulence gene expression was taken as dichotomous variables (*i.e*. present or absent). Kendall's tau-b and Spearman's rho correlation tests were employed in IBM SPSS version 23.0 (IBM, 2015) to identify monotonic relationships in non-normally distributed datasets. Additionally, One-Sample Binomial Test was conducted to assess whether the observed prevalence of virulence significantly differed from a hypothesized proportion of 50%.

### 2.15 Preparation of outer membrane protein antigen (OMP)

The outer membrane protein (OMP) from *Salmonella* spp. was prepared following Maji et al. (2006) with modifications. In brief, *Salmonella* spp. strains were cultured in Nutrient broth (Hi-Media, India) at 37 °C for 48 h. The grown culture underwent heat killing at 80 °C for 1 hour. Bacterial cells were harvested by centrifugation at 1,500 g for 40 min at room temperature. Cell pellets were washed, re-suspended in 7.8 ml sterile NSS (NaCl, Hi-Media, India) with 2% SDS (Hi-Media, India) and 2-mercaptoethanol (Hi-Media, India), and treated at 60 °C for 1 h for solubilisation. Solubilized extracts (OMP) were centrifuged at 1,500 g for 60 min at room temperature, filtered (using 0.22 μ membrane), and stored at −20 °C as crude OMP antigen. Protein concentration was estimated using the Lowry's method (Lowry et al., 1951).

### 2.16 Characterization of OMP by sodium dodecyl sulphate polyacrylamide gel electrophoresis (SDS-PAGE)

Protein samples were analyzed using one-dimensional SDS-PAGE in a vertical slab gel electrophoresis system (Atto, Japan) following Laemmli (1970) with some modifications. In brief, 12.5% polyacrylamide gel (15 x 17 cm) was prepared. Protein samples (25 μl mixed with buffer 1:1) were heated at 100 °C for 5 min, and 50 μg of protein was loaded per well, with 5 μl of molecular weight marker in one well. Electrophoresis was conducted at 20 mA for approximately 4 h. The gel was stained with 0.1% Coomassiebrilliant blue staining solution overnight, destained by destaining solution containing methanol and acetic acid.

### 2.17 Preparation of hyperimmune serum in rabbit

Hyperimmune serum against the crude outer membrane protein (OMP) antigen of *Salmonella* C-10a was raised in a rabbit, following Sahoo and Joardar (2004) with some modifications. Briefly, a healthy New Zealand white male rabbit weighing 900 g, received intramuscular injections (gluteal muscle) of *Salmonella* OMP antigen mixed with an equal volume of Freund's adjuvant (Genei, India) at 7-day intervals. The antigen doses ranged from 800 μg to 1,400 μg per injection. Five days after the final injection, blood was collected from the rabbit, and the serum was separated and stored at −20 °C. Serum collected from another rabbit of same breed, sex and weight was used as a control.

### 2.18 Counter-current immuno-electrophoresis

Counter Current Immuno-Electrophoresis was performed in 1.5% agarose prepared in 0.5M Tris Borate Buffer (pH 8.6). For the immuno-electrophoresis, 10 μl of *Salmonella* OMP antigen, and 10 μl of hyperimmune serum was used. A current of 2 mA was applied for 1 h.

### 2.19 Enzyme linked immunosorbent assay (ELISA)

The enzyme linked immunosorbent assay (ELISA) was carried out in a 96-well plate following Sahoo and Joardar (2004). In brief, *Salmonella* OMP antigen was coated in the plate overnight at 4 °C. Blocking buffer (5% skimmed milk powder with 2% gelatine in PBS, 200 μl) was added to all wells and incubated at 37 °C for 2 h, followed by washingwith PBS-T. The wells were then incubated at 37 °C for 2 h with appropriately diluted 100 μl of each negative control serum and test (hyperimmune) serum, followed by three washes with PBS-T. Anti-rabbit conjugate (diluted 1,000 times) was added (100 μl/well) and incubated at 37 °C for 2 h. Following washing, substrate solution (100 μl) was added. Finally, 50 μl of 3N H_2_SO_4_ solution was added to stop the reaction.

### 2.20 Western blot

Proteins were separated by SDS-PAGE (22) and transferred electrophoretically onto a nitrocellulose membrane (NCM) using a semi-dry electroblotter (Atto, Japan) as per Towbin et al. (1979) with some modifications. In brief, complete transfer of proteins from gel was achieved in 90 min at a current of 2 mA/cm^2^ of gel. The NCM was then blocked overnight at 4 °C using PBS (pH 7.2) having 5% bovine serum albumin. After washing with PBS-T buffer the membrane was incubated with a 1:20 dilution of hyperimmune serum in dilution buffer for 2 h. After washing, the NCM was incubated with Rabbit anti-goat conjugate (1:500 dilutions). Finally, the blot was developed using substrate solution (Tris-HCl, H_2_O_2_, and Di-aminobenzidine tetrahydrochrloride (DAB.4HCl).

### 2.21 Dot ELISA

Dot ELISA was performed as per Adikesavalu et al. (2016) with some modifications. In brief, Nitrocellulose membranes were cut into strips, with two strips for each antigen (one test and one control), in total twelve strips for six antigens. Each strip was coated with 50μl of antigen and incubated at 37 °C for 2 h. These were blocked overnight. Control and test sera (50μl each) were added to the strips and incubated at 37 °C for 2 h. Diluted (1:1000) Goat anti-rabbit IgG-HRPO conjugate (Genei, India) was used as secondary immunoconjugate and DAB–H_2_O_2_ substrate for color development.

## 3 Results

### 3.1 Detection and characterization of *Salmonella* from cloacal swabs, tracheal swabs and environmental samples

**Figure 2:**
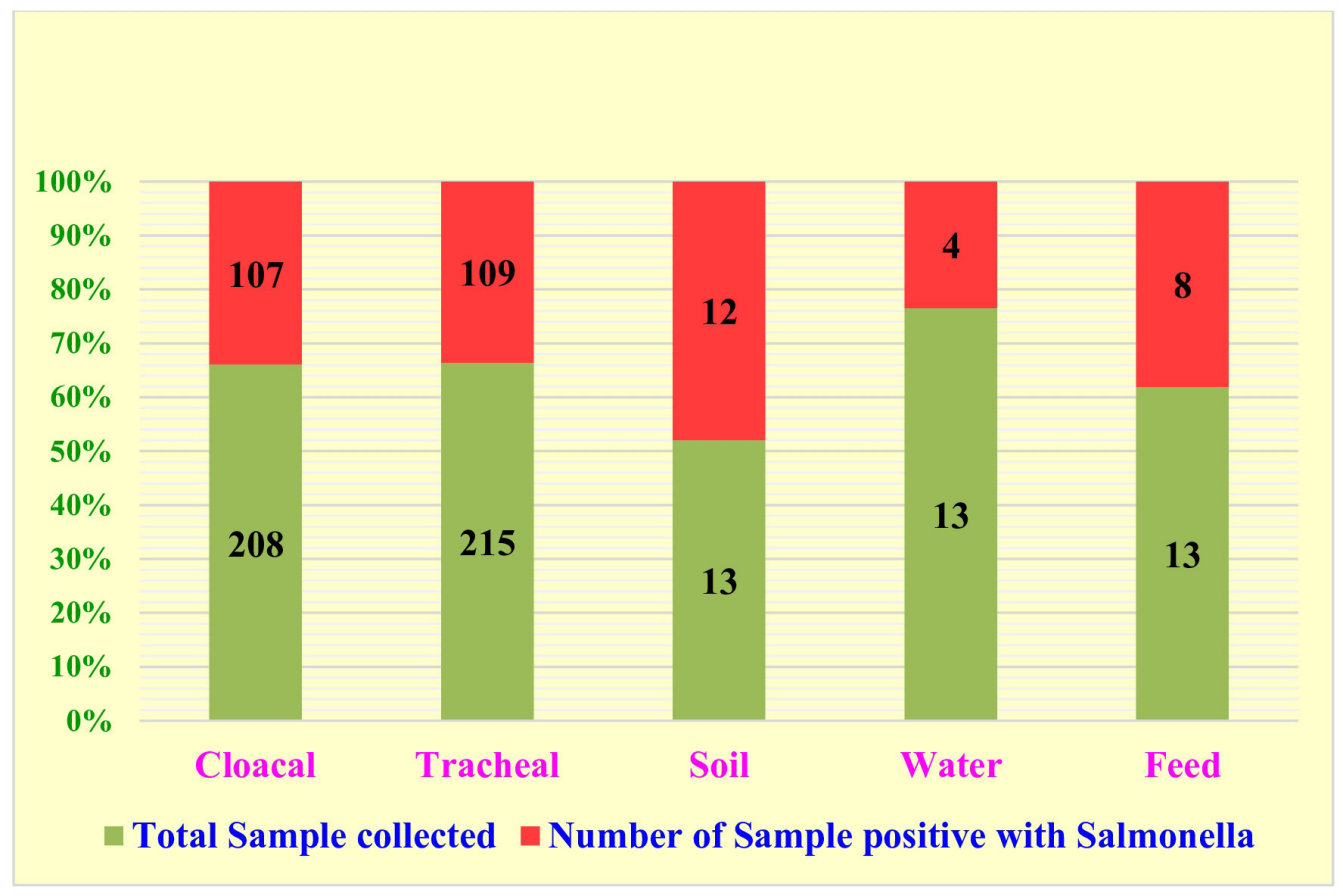
Distribution of *Salmonella* isolated from cloacal, tracheal and environmental sample.

Out of 462 samples analyzed, 240 exhibited typical characteristics of *Salmonella* spp. in Selenite F broth and Xylose Lysine Deoxycholate (XLD) Agar. Analysis revealed 51.95% (240/462) occurrence of *Salmonella* spp., nearly equal rates in cloacal swabs (51.44%, 107/208) and tracheal swabs (50.70%, 109/215). Environmental samples showed varied prevalence: 61.54% (8/13) in feed, 30.77% (4/13) in water, and 92.31% (12/13) in soil. From these 240 positive samples, a total of 436 *Salmonella* spp. isolates were generated based on distinct single colonies formed in the plates, predominantly from cloacal swabs (45.41%, 198/436) and tracheal swabs (43.58%, 190/436), with fewer from environmental samples (11.01%, 48/436) (Figure 2).

### 3.2 Detecting extended spectrum β-lactamase (ESBL) in *Salmonella* isolates through double-disc assay for phenotypic characterization

Among the 436 *Salmonella* spp. isolates tested, 184 (42.20%) were found to be ESBL producers based on double disc synergy assay.

### 3.3 Detecting extended spectrum β-lactamase (ESBL) and *AmpC* β-lactamase (ACBL) in *Salmonella* spp. isolates through PCR

The *bla*_*CTX*−*M*_gene was present in 90 (20.64%) isolates, distributed as 32.22% (29 isolates) from cloacal swabs, 55.56% (50 isolates) from tracheal swabs, and 12.22% (11 isolates) from environmental samples. The *bla*_*SHV*_ gene was positive in 77 (17.66%) of the 436 isolates tested. These isolates were sourced as follows: 18.18% (14 isolates) from cloacal swabs, 68.83% (53 isolates) from tracheal swabs, and 12.99% (10 isolates) from environmental samples. A total of 159 (36.47%) isolates were found positive for the *bla*_*TEM*_ gene, distributed as 42.77% (68 isolates) from cloacal swabs, 47.80% (76 isolates) from tracheal swabs, and 9.43% (15 isolates) from environmental samples. Further, the *bla*_*AmpC*_gene was observed in 142 (32.57%) isolates, with a distribution of 32.39% (46 isolates) from cloacal swabs, 55.63% (79 isolates) from tracheal swabs, and 11.97% (17 isolates) from environmental samples.

The nucleotide sequences of the PCR products were compared and found similar with *bla*_*CTX*−*M*−15_, *bla*_*CTX*−*M*−28_, *bla*_*CTX*−*M*−82_, *bla*_*SHV*−215_, *bla*_*SHV*−27_, *bla*_*SHV*−45_, *bla*_*SHV*−191_, *bla*_*SHV*−2_, *bla*_*SHV*−249_, *bla*_*SHV*−99_, *bla*_*TEM*−72_, *bla*_*TEM*−1_, and *bla*_*TEM*−164_ in the BLAST search. The sequences were published by DNA Data Bank of Japan (DDBJ) with accession numbers (https://getentry.ddbj.nig.ac.jp/) (Table 2).

**Table 2:** Accession numbers of nucleotide sequences of ESBL genes possessed by *Salmonella* sp. strains isolated from Duck.

**Bacteria**	**ESBL type**	**Source**	**Strain No**.	**Place**	**Accession Number**
*Salmonella* sp.	SHV-215	Duck Trachea	SPB1	PurbaBardhaman	LC774610
*Salmonella* sp.	SHV-27	Duck Trachea	SM2	Mohanpur	LC774700
*Salmonella* sp.	SHV-45	Duck Trachea	Sal_T-21b_Ban	Bankura	LC876923
*Salmonella* sp.	SHV-215	Duck Feed	Sal_BF-2a_Bar	Barasat	LC876924
*Salmonella* sp.	SHV-191	Duck Trachea	Sal_T-10C_Ban	Bankura	LC875420
*Salmonella* sp.	SHV-2	Duck Cloaca	Sal_C-7c_Bard	PurbaBardhaman	LC876679
*Salmonella* sp.	SHV-249	Duck Cloaca	Sal_C-35a_Moh	Mohanpur	LC876680
*Salmonella* sp.	SHV-99	Duck Trachea	Sal_T-4b_Ban	Bankura	LC876681
*Salmonella* sp.	CTX-M-15	Duck Cloaca	SBAN2	Bankura	LC774654
*Salmonella* sp.	CTX-M-28	Duck Cloaca	Sal_C-10a_Bard	PurbaBardhaman	LC874602
*Salmonella* sp.	CTX-M-15	Duck Trachea	Sal_T-7b_Moh	Mohanpur	LC874701
*Salmonella* sp.	CTX-M-15	Duck Trachea	Sal_T-18b_Moh	Mohanpur	LC874702
*Salmonella* sp.	CTX-M-82	Duck Cloaca	Sal_C-6d_Bard	PurbaBardhaman	LC875419
*Salmonella* sp.	TEM-72	Duck Cloaca	Sal_C-6d_Bard	PurbaBardhaman	LC878793
*Salmonella* sp.	TEM-1	Duck Cloaca	Sal_C-10a_Bard	PurbaBardhaman	LC878794
*Salmonella* sp.	TEM-164	Duck Trachea	Sal_T-10c_Bard	PurbaBardhaman	LC878795
*Salmonella* sp.	TEM-72	Duck Feed	Sal_BF-2a_Bar	Barasat	LC878796

### 3.4 Culture plate method for identifying biofilm-producing *Salmonella* spp. isolates.

The tissue culture plate method using crystal violet dye showed a substantial proportion of the bacterial isolates with considerable potential for biofilm formation, displaying varying degrees of adherence. Among the tested *Salmonella* spp. isolates, 20.41% (89) demonstrated robust biofilm-forming capabilities, 39.91% (174) displayed moderate biofilm formation, and 27.52% (120) exhibited weak biofilm production, while 12.16% (53) of isolates showed no biofilm formation.

### 3.5 PCR-based identification of biofilm-associated genes in *Salmonella* spp. isolates

The investigation focused on four specific genes associated with biofilm formation: *csgA, rcsA, rpoS*, and *sdiA*. A total of 238 isolates (54.59%) were positive for the *csgA* gene. These included 117 isolates (49.16%) from cloacal swabs, 102 isolates (42.86%) from tracheal swabs, and 19 isolates (7.98%) from environmental samples. For the *sdiA* gene, 229 (52.52%) isolates tested positive. Of these, 101 isolates (44.10%) were from cloacal swabs, 104 isolates (45.42%) from tracheal swabs, and 24 isolates (10.48%) from environmental samples. The *rpoS* gene was present in 350 (80.28%) of the isolates, with 156 (44.57%) from cloacal swabs, 158 (45.14%) from tracheal swabs, and 36 (10.29%) from environmental samples. Moreover, the *rcsA* gene was detected in 278 (63.76%) of the isolates. The distribution was 128 (46.04%) from cloacal swabs, 121 (43.53%) from tracheal swabs, and 29 (10.43%) from environmental samples.

### 3.6 Detection of virulence gene *invA* in isolated *Salmonella*

It was found that 141 (32.34%) isolates were positive for the *invA* gene. Among these, 43.97% (62 isolates) were from cloacal swabs, 48.23% (68 isolates) from tracheal swabs, and 7.80% (11 isolates) from environmental samples.

### 3.7 Detection of common traits in *Salmonella* isolates

A total of 26 *Salmonella* isolates revealed the coexistence of three essential traits: ESBL production, biofilm-forming ability, and virulence gene expression. Within this group, only 6 isolates were positive for each of the ESBL, biofilm, and virulence genes tested. These isolates, identified as C-10a (from Purba Bardhaman), C-6d (from Purba Bardhaman), T-7b (from Mohanpur), T-18b (from Mohanpur), T-10c (from Bankura), and BF-2a (from Barasat), were selected for outer membrane protein extraction.

### 3.8 Comparative *in vitro* analysis of antibiotic sensitivity (ABST) among *Salmonella* isolates having ESBL, biofilm and virulence gene

**Figure 3:**
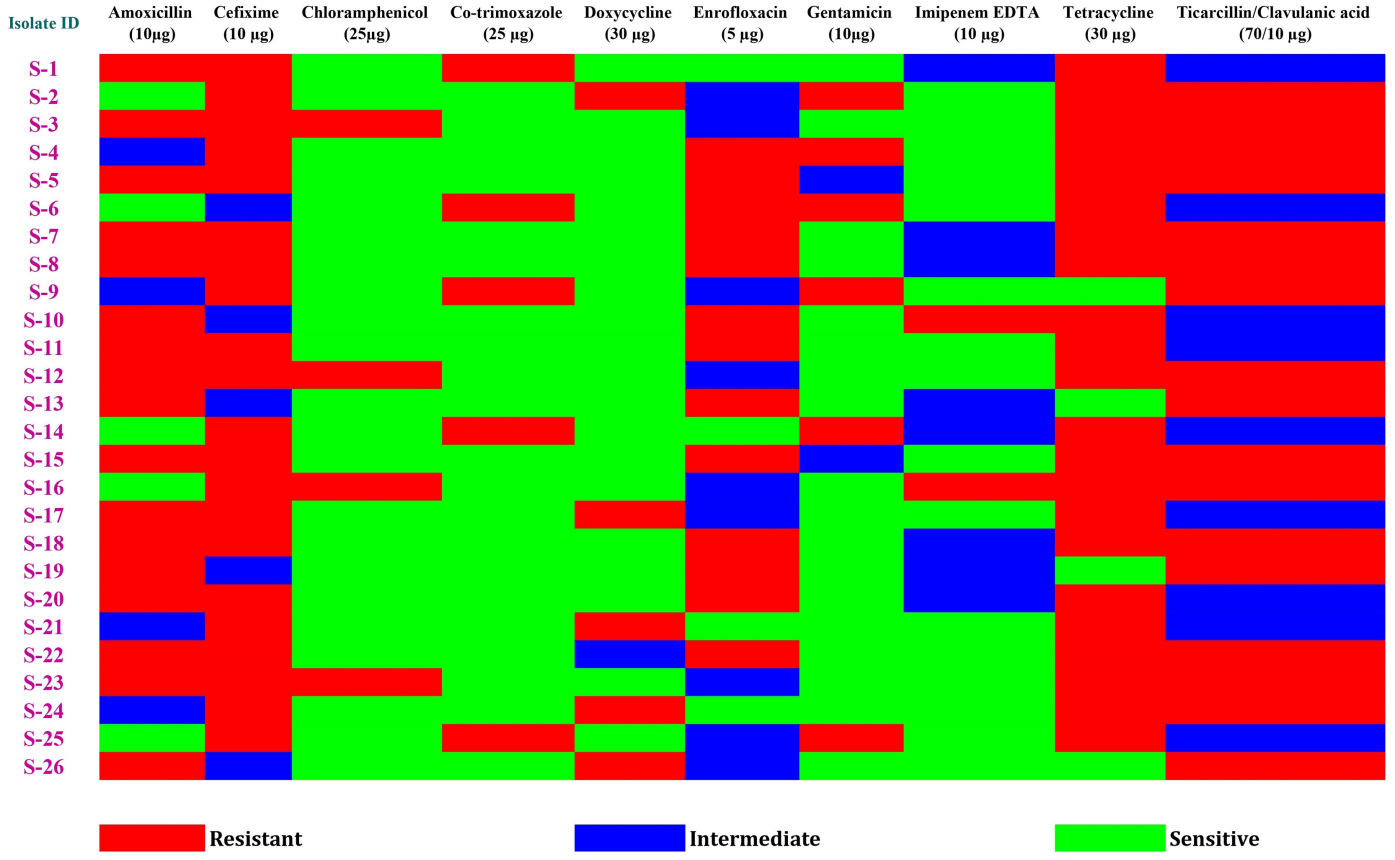
Heatmap showing phenotypical antibiotic resistance profile of *Salmonella* strains with three essential traits: ESBL production, biofilm forming ability, and virulence gene expression isolated from Duck and their associated environment from West Bengal (India).

The study specifically targeted 26 *Salmonella* spp. isolates for antibiotics sensitivity testing (Figure 3), revealing the coexistence of three essential traits: ESBL production, biofilm forming ability, and virulence gene expression within this group. Tetracycline (30 μg) exhibited the highest resistance among the *Salmonella* spp. isolates, with 84.62% showing resistance, followed closely by Cefixime (10 μg) at 80.77%. Amoxicillin (10 μg) and Ticarcillin/Clavulanic acid (70/10μg) displayed a resistance rate of 65.39%, while Enrofloxacin (5 μg) had a resistance rate of 50%. On the other hand, Chloramphenicol (25 μg) displayed sensitivity in 84.62%, Co-trimoxazole (25 μg) in 80.77%, Doxycycline (30 μg) in 76.92%, Gentamicin (10 μg) in 69.23%, and Imipenem EDTA (10 μg) in 61.54% of instances, establishing them as the most potent antibiotics against the *Salmonella* spp. isolates (Table 3).

**Table 3:** Antibiotic Sensitivity Test of *Salmonella* isolates (n=26) having ESBL, biofilm and virulence genes.

**Sl. No**.	**Antibiotics (Concentration)**	**Sensitive (%)**	**Intermediate (%)**	**Resistant (%)**
01	Amoxicillin (10μg)	05 (19.22%)	04 (15.39%)	17 (65.39%)
02	Cefixime (10 μg)	00	05 (19.23%)	21 (80.77%)
03	Chloramphenicol (25μg)	22 (84.62%)	00	04 (15.38%)
04	Co-trimoxazole (25 μg)	21 (80.77%)	00	05 (19.23%)
05	Doxycycline (30 μg)	20 (76.92%)	01 (3.85%)	05 (19.23%)
06	Enrofloxacin (5 μg)	04 (15.38%)	09 (34.62%)	13 (50%)
07	Gentamicin (10 μg)	18 (69.23%)	02 (7.69%)	06 (23.08%)
08	Imipenem EDTA (10 μg)	16 (61.54%)	08 (30.77%)	02 (7.69%)
09	Tetracycline (30 μg)	04 (15.38%)	00	22 (84.62%)
10	Ticarcillin/Clavulanic acid (70/10 μg)	00	09 (34.61%)	17 (65.39%)

### 3.9 Phylogenetic analysis by dendrogram

**Figure 4:**
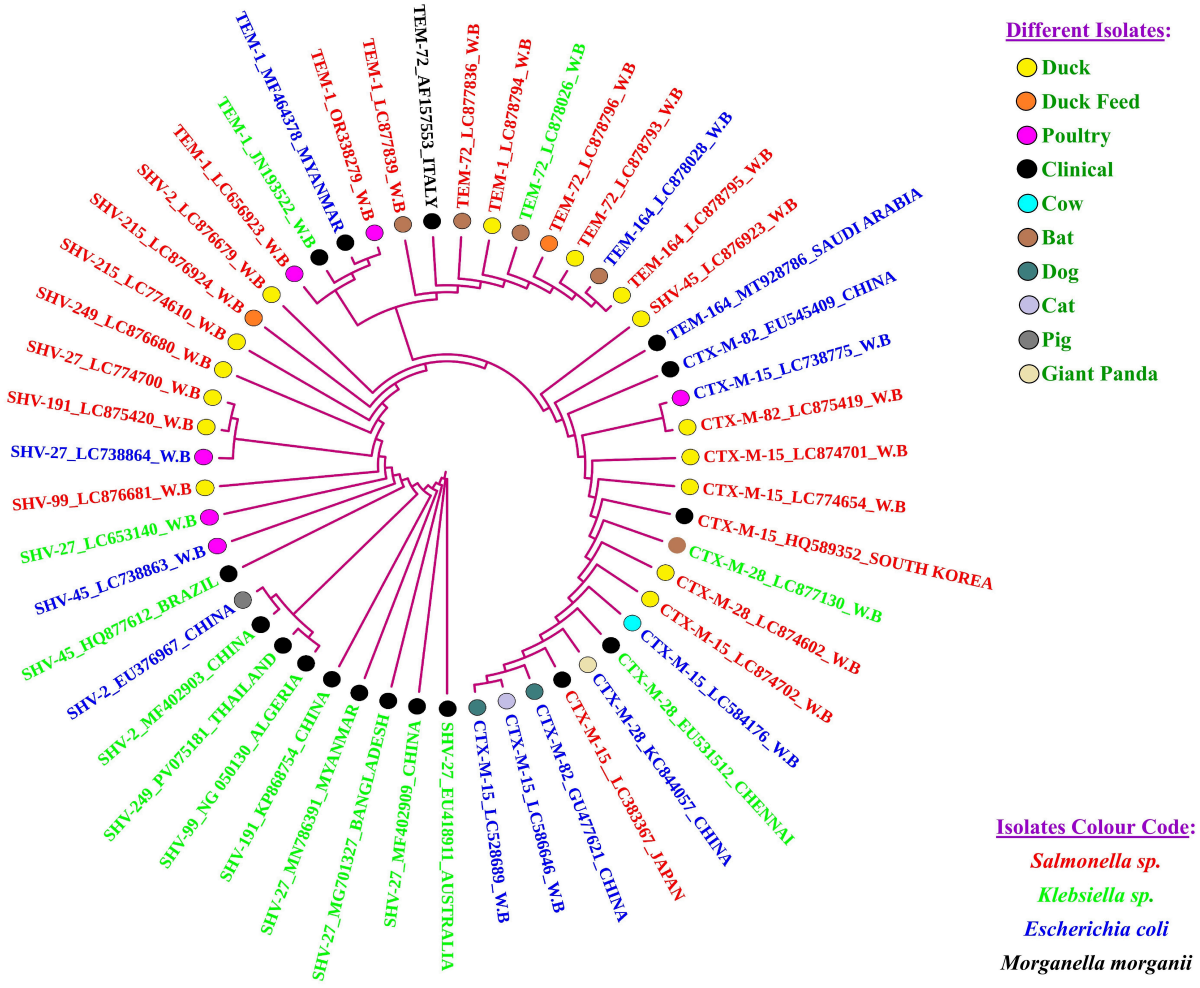
Clonal relationship of β-lactamase-producing *Salmonella* isolatedfrom Duck and their associated environment in west Bengal (India) with human clinical isolates, poultry, cow, dog, cat, bat, pig and giant panda.

On phylogenetic analysis, a partial clonal relationship of β-lactamase gene sequences identified in this study, viz, three *bla*_*CTX*−*M*−15_(LC774654, LC874701, LC874702), one *bla*_*CTX*−*M*−28_(LC874602), one *bla*_*CTX*−*M*−82_(LC875419), two *bla*_*SHV*−215_(LC774610, LC876924), one *bla*_*SHV*−27_(LC774700), one *bla*_*SHV*−45_(LC876923), one *bla*_*SHV*−191_ (LC875420), one *bla*_*SHV*−2_(LC876679), one *bla*_*SHV*−249_(*LC876680*), one *bla*_*SHV*−99_(LC876681), two *bla*_*TEM*−72_(LC878793, LC878796), one *bla*_*TEM*−1_(LC878794), and one *bla*_*TEM*−164_(LC878795) with sequences from human clinical isolates, poultry, cow, dog, cat, bat, pig, and giant panda (Figure 4). These related isolates were reported from various global regions including the Asian subcontinent (India, Bangladesh, China, Japan, Myanmar, Saudi Arabia, South Korea, and Thailand), the American subcontinent (Brazil), the African subcontinent (Algeria), the European subcontinent (Italy), and Oceania (Australia), indicating a widespread distribution and possible interspecies dissemination.

### 3.10 Association study by Statistical analysis

Statistical analysis revealed highly significant (*p* < 0.001) positive associations among biofilm formation, antibiotic resistance, and virulence gene (*invA*) prevalence in *Salmonella* isolates. A strong correlation was observed between biofilm formation and antibiotic resistance (τ = 0.656, ρ = 0.765; *p* < 0.001), indicating that the isolates with enhanced biofilm-forming ability, exhibited greater levels of antimicrobial resistance. Additionally, a moderate correlation was revealed between antibiotic resistance and virulence (τ = 0.398, ρ = 0.430; *p* < 0.001), while a weaker but highly significant association was noted between biofilm formation and virulence (τ = 0.270, ρ = 0.297; *p* < 0.001).

### 3.11 Proteins of the outer membrane of *Salmonella* isolate

The concentrations of OMP (outer membrane proteins) in the characterized *Salmonella* are given in the Table 4.

**Table 4:** Concentrations of the Outer membrane protein (OMP) as assessed by Lowry's method.

**Sl No**.	***Salmonella* spp. OMP**	**O.D._660_**	**Concentration (mg/ml)**
1.	Standard	0.3161	1.000
2.	C-10a	1.1019	3.486
3.	C-6d	0.2863	0.906
4.	T-7b	0.1575	0.498
5.	T-18b	1.2687	4.014
6.	T-10c	0.2673	0.846
7.	BF-2a	0.5330	1.686

### 3.12 Comprehensive Analysis of outer membrane proteins of ESBL-Producing, biofilm forming and virulence gene bearing *Salmonella* spp. isolates by SDS-PAGE

**Figure 5:**
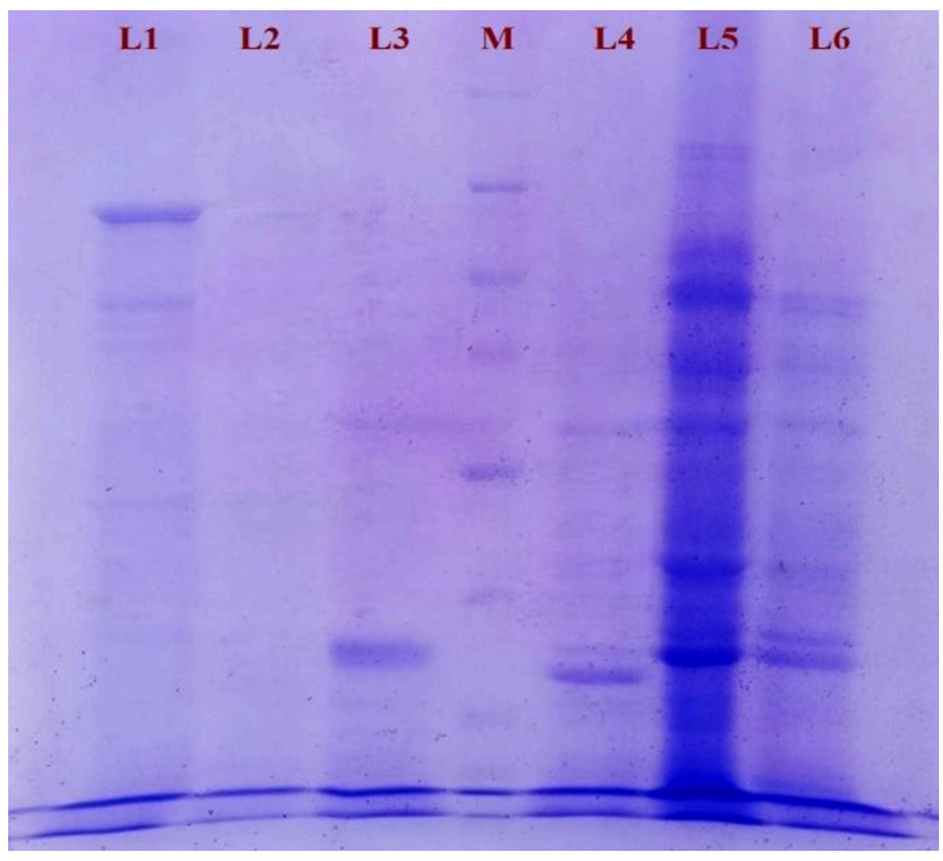
Polypeptide profile of the outer membrane proteins of *Salmonella* spp. isolates as assessed by SDS-PAGE [L1: C-6d OMP, L2: T-7b OMP, L3: T-18b OMP, M: ProteinMarker, L4: T-10c OMP, L5: C-10a OMP, L6: BF-2a OMP].

The analysis of outer membrane proteins (OMPs) from six distinct *Salmonella* spp. isolates revealed a diverse and variable banding pattern when subjected to SDS-PAGE analysis. The molecular weights of these bands ranged from 5 kDa to 109 kDa, highlighting the complex proteomic profile of OMPs within these isolates. Among the isolates examined, the C-6d strain exhibited the presence of 15 distinct protein bands, indicating a relatively diverse range of OMPs within this isolate. Conversely, T-7b and T-18b strains displayed a simpler OMP profile with only 8 bands. T-10c and BF-2a strains, on the other hand, exhibited 14 and 17 bands, respectively, indicating diverse complexity in their OMP composition. Of particular interest, the C-10a strain demonstrated the highest number of protein bands, a total of 21 bandswith molecular masses between 9 and 109 kDa. The molecular masses of 21 predominant proteins were calculated as 109, 105, 99, 95, 93, 89, 85, 78, 75, 66, 51, 48, 45, 41, 39, 35, 33, 30, 20, 15 and 9 kDa (Figure 5).

### 3.13 Validation of hyper-immunized serum by counter current immuno-electrophoresis analysis (CCIE)

The distinct precipitin line between the wells indicated presence of specific antibodies against the *Salmonella* OMP antigen in the tested (hyper-immune) serum.

### 3.14 Assessment of immunogenicity of *Salmonella* spp. outer membrane protein (OMP) by indirect enzyme-linked immunosorbent assay (i-ELISA)

The hyperimmune serum showed elevated antibody responses (in terms of OD values) against all OMPs compared to the control serum values. The antibody titre in the hyperimmunized serum was assessed through serial dilution, starting at 1:100 and progressing to 1:204800. The highest dilution with 50% of the maximum absorbance was 1:12800, indicating an antibody titre of 12800.

### 3.15 Assessment of immunoreactivity of *Salmonella* spp. outer membrane proteins by western-blotting analysis

**Figure 6:**
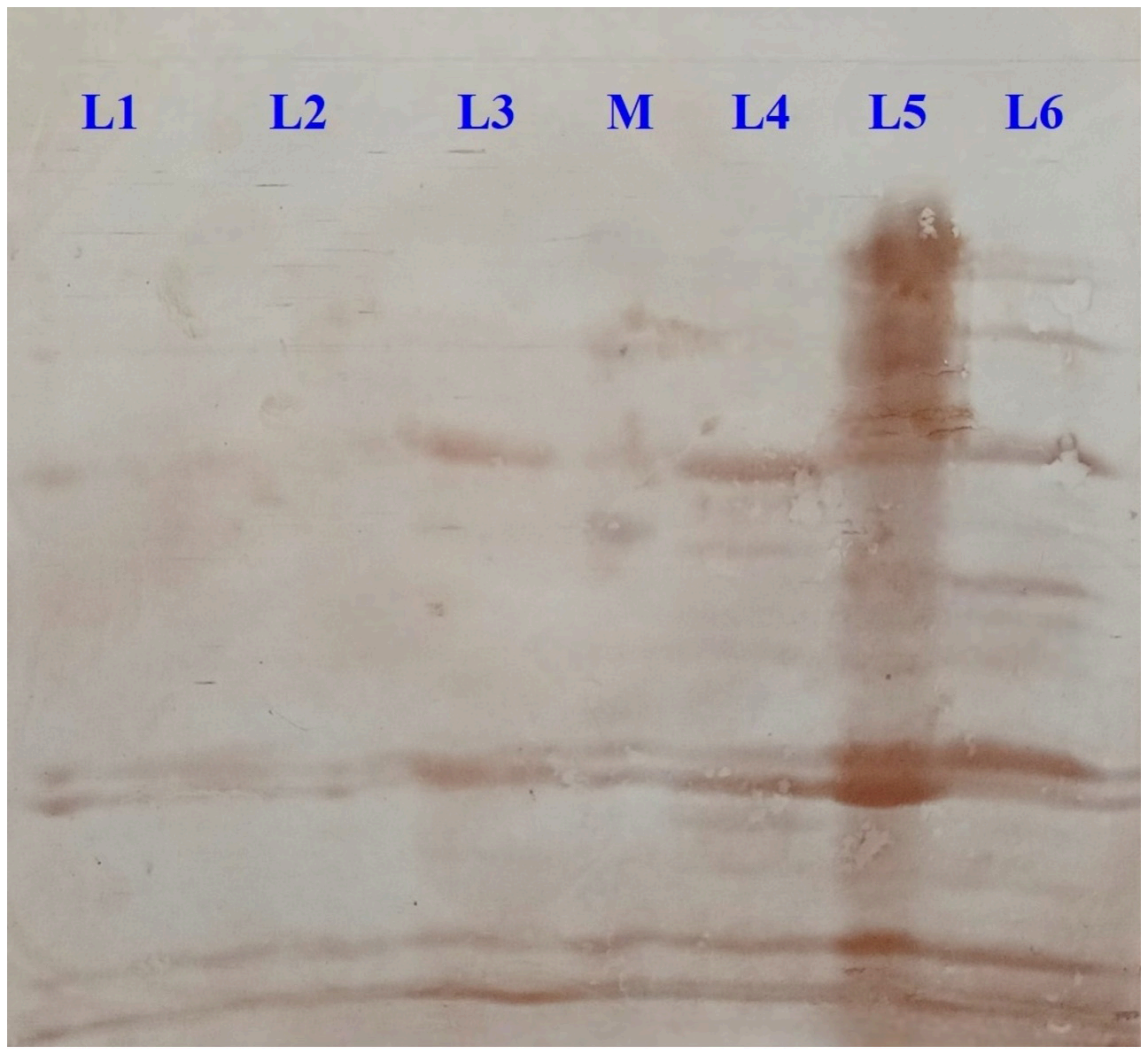
Western blot analysis showing immunoreactivity of crude outer membrane proteins (OMPs) derived from *Salmonella* spp. [L1: C-6d OMP, L2: T-7b OMP, L3: T-18b OMP, M: Protein Marker, L4: T-10c OMP, L5: C-10a OMP, L6: BF-2a OMP].

Significant immunoreactivity was observed in four out of six selected OMPs. Specifically, the T-18b OMP showed immunoreactivity at 69 and 35 kDa, while the T-10c OMP exhibited reactivity at 69, 35, and 33 kDa. The C-10a OMP demonstrated the highest intensity of immunoreactivity with bands at 85, 75, 69, 35, 33, and 20 kDa. The BF-2a OMP showed reactivity at 69, 51, 44, 35, and 33 kDa (Figure 6).

### 3.16 Evaluation of seroreactivity to *Salmonella* spp. outer membrane protein (OMP) antigen via DOT ELISA

Clear dot was observed in the test NCP strip with serum from the rabbit, experimentally inoculated with OMP of *Salmonella* spp. carrying antibiotic resistance (ESBL), virulence, and biofilm genes, indicating the presence of anti-*Salmonella* antibodies. However, no color developed in control serum.

## 4 Discussion

Duck meat as a source of food-borne infection is an emerging zoonotic hazard and the studies revealed that in total 2% of global food-borne outbreaks is associated with consumption of undercooked duck meat (Ljubojević Pelić et al., 2021). *Salmonella*, the major meat-borne pathogen causing outbreaks throughout the world, was found as the major contaminant (29–51%) of duck meat even more than the poultry (3.7–5%) and turkey (5%) (Little et al., 2008; Yoon et al., 2014). High nutritional value and delicious taste are the major attraction of the duck meat for the consumers world-wide which in turn provide a good return to the duck rearing farmers. It is crucial to diagnose virulent *Salmonella*, especially with biofilm and antibiotic resistance properties, to preserve the safety of the duck meat.

The prevalence of *Salmonella* spp. in duck samples is a critical issue for food safety and public health as under-cooked duck meat and egg are used in preparation of meat and egg based products. In this study, *Salmonella* spp. were identified in 51.95% of the samples analyzed (240/462). Among these, cloacal swabs accounted for 51.44% (107/208), tracheal swabs 50.70% (109/215), and environmental samples 61.54% (24/39). These findings indicate significant contamination even in apparently healthy ducks, highlighting the potential for environmental contamination within duck-raising environments. The prevalence of *Salmonella* spp. observed in this study (51.95%) contrasts significantly with previous findings. Mir et al. (2015) reported a much lower prevalence of 6.31% in fecal and caecal contents of ducks, chickens, and emus. Harsha et al. (2011) found 6% prevalence in duck eggshells and a higher 51.33% in egg contents in Kerala. Similarly, Mondal et al. (2008) noted 13.07% prevalence in duck cloacal swabs in Bangladesh, while Nor Faiza et al. (2013) reported 16% prevalence in cloacal swabs and none in duck eggs in Malaysia. In contrast, Olaitan et al. (2011) and Adzitey et al. (2012) documented higher prevalence rates of 30.5% and 23.54%, respectively. Earlier study reported 39.58% prevalence in ducks from the Dinajpur district of Bangladesh (Rahman et al., 2016). These variations in prevalence rates may be due to differences in sampling methodologies, timeframes, and regional differences in duck management and environmental conditions. The strikingly high prevalence of *Salmonella* detected in soil samples (92.31%) highlights the farm environment as a critical reservoir for pathogen persistence. *Salmonella* is known to survive for extended periods in soil and manure-amended environments, with survival influenced by soil type, temperature, and organic matter content, which supports its persistence outside the host (survival in manure-amended soils) (Phan-Thien et al., 2020). Environmental reservoirs such as soil and dust have been implicated in ongoing contamination and horizontal transmission of *Salmonella* in poultry production systems, where feces, dust, and aerosols can contribute to pathogen dissemination and sustained infection pressure within flocks (Gast et al., 2025). The comparatively high occurrence of *bla*_*AmpC*_ and *bla*_*SHV*_ genes in isolates recovered from tracheal swabs points toward a different exposure or colonization route potentially involving the respiratory tract. Airborne dust has been shown experimentally to transmit *Salmonella* and facilitate colonization, mimicking field conditions of absorption via inhaled particles in poultry systems (Khan et al., 2024). Taken together, these observations suggest the coexistence of two interconnected contamination pathways: one driven by environmental persistence and another linked to respiratory exposure, both of which may play an important role in the circulation and spread of antimicrobial-resistant *Salmonella* within duck production settings.

The relationship between environmental contamination and *Salmonella* carriage in ducks is likely bidirectional. In semi-intensive duck farming systems, infected or asymptomatic carrier ducks continuously shed *Salmonella* through feces, leading to contamination of soil, water, and feed (Foley et al., 2011; Davies and Wales, 2010). Once established in the environment, *Salmonella* can persist for prolonged periods and serve as a constant source of re-exposure, thereby sustaining intestinal and, potentially, respiratory carriage within the flock (Whiley and Ross, 2015; Liu et al., 2018). This environmental amplification cycle is particularly relevant for dominant serovars such as *Salmonella Typhimurium* and *Salmonella Enteritidis*, which are well adapted to both host colonization and environmental survival. From a public health perspective, the circulation of these serovars is of concern, as they are among the most frequently implicated in human salmonellosis worldwide and are commonly associated with multidrug resistance (Crump et al., 2015). The presence of antimicrobial-resistant strains in both environmental and host-associated samples suggests that duck farming environments may act as important reservoirs facilitating persistence, dissemination, and potential spillover of resistant *Salmonella* strains along the food chain, thereby posing a risk to both animal and human health.

The majority of ESBL-producing bacteria in poultry are from the *E. coli* and *Salmonella* groups, as reported worldwide (Saliu et al., 2017). In the present study, ß-lactamase production was detected in 42.20% (184) *Salmonella* spp. isolates when phenotypic confirmation of the presence of ESBLs was assessed. The study also examined the occurrence of extended-spectrum β-lactamase (ESBL) genes in the isolates. The results showed that 36.47% of the isolates harbored the *bla*_*TEM*_ gene, 20.64% harbored *bla*_*CTX*−*M*_, 17.66% harbored *bla*_*SHV*_, and 32.57% carried *bla*_*AmpC*_ genes. This highlights the significant presence of antibiotic resistance genes in *Salmonella* spp. from duck samples. The findings of this study align with previous research on ESBL genes in *Salmonella* spp. Banerjee et al. (2019) identified *bla*_*CTX*−*M*_in 5 isolates and a higher prevalence of *bla*_*AmpC*_ (46.15%) compared to our results. Conversely, Zhang et al. (2018) reported 21 ESBL-producing isolates from 615 samples, including *bla*_*CTX*−*M*_-55, *bla*_*CTX*−*M*_-123, *bla*_*TEM*_-206, and *bla*_*TEM*_-214, but no *bla*_*SHV*_. Bai et al. (2015) found *bla*_*CTX*−*M*_and *bla*_*TEM*_ in 11 and 5 isolates, respectively, in *Salmonella*enteric serovar Indiana from chicken and pig meat.

The occurrence of ESBL producers in studied duck population was observed in absence of therapeutic or non-therapeutic exposure (no commercial feed used) to the higher generation cephalosporins. Probably the occurrence was correlated with the exposure to contaminated environment during daytime roaming of the ducks (Banerjee et al., 2019).

Furthermore, the investigation of virulence genes revealed that 32.34% of the isolates were positive for the *invA* gene. Among these *invA*-positive isolates, 43.97% were from cloacal swabs, 48.23% from tracheal swabs, and 7.80% from environmental samples. This prevalence of virulence genes in the isolates suggests a potential risk for severe infections. In comparison, Salehi et al. (2005) detected *Salmonella* in 15.6% of poultry carcasses via the *invA*gene, which is considerably lower than our study's prevalence. Osman et al. (2014) identified 18.5% *Salmonella* isolates from imported ducklings, all positive for the *invA* gene, again lower than our findings. Staji et al. (2017) found 7.2% of *Salmonella enterica* strains in mallard duck fecal samples, with 50% positive for *invA*, also lower than our results. Ammar et al. (2016), Mir et al. (2010), and Krawiec et al. (2015) reported 17%, 6.88%, and 6.4% prevalence rates of *invA*-positive *Salmonella* isolates, respectively, all lower than in this study. These variations can be attributed to differences in study populations, sample sources, geographical locations, methodologies, and bacterial strain diversity. The study also analyzed the presence of biofilm-forming genes (*csgA, sdiA, rpoS*, and *rcsA*) in *Salmonella* spp. isolates. The results showed that 54.59% of the isolates harboured*csgA*, 52.52% harboured*sdiA*, 80.28% carried *rpoS*, and 63.76% carried *rcsA* genes. This indicates a high potential for biofilm formation among the isolates, which can contribute to their persistence and resistance in the environment. Agarwal et al. (2011) reported that 57.61% of *Salmonella* spp. isolates were moderate biofilm producers, which is higher than the current study's figures. They found 22.5% and 19.21% were weak and strong biofilm producers, respectively, lower than the current findings. Ahmad et al. (2020) recovered 90 *Salmonella* isolates from deceased poultry birds' liver samples in Peshawar, Pakistan, and observed 52.94% strong, 39.02% moderate, 7.31% weak, and 1.21% non-biofilm formers using microtitre plate assays. Comparatively, our study noted fewer strong biofilm formers, similar rates of moderate biofilm formers, weaker biofilm formers, and a higher presence of non-biofilm formers than Ahmad et al. (2020).

In this study, a subset of 26 *Salmonella* spp. isolates—each exhibiting ESBL production, biofilm-forming ability, and virulence gene expression—revealed a concerning trend in antibiotic susceptibility patterns. The highest resistance was observed against Tetracycline (30 μg) at 84.62%, followed closely by Cefixime (10 μg) at 80.77%. Resistance to Amoxicillin (10 μg) and Ticarcillin/Clavulanic acid (70/10 μg) was also notable, each at 65.39%, while Enrofloxacin (5 μg) showed 50% resistance. In contrast, the highest sensitivity was seen with Chloramphenicol (25 μg), effective against 84.62% of isolates, followed by Co-trimoxazole (25 μg) at 80.77%, Doxycycline (30 μg) at 76.92%, Gentamicin (10 μg) at 69.23%, and Imipenem EDTA (10 μg) at 61.54%, identifying these as the most effective agents. The elevated prevalence of tetracycline resistance observed in this study aligns with previous reports from Bangladesh (Das et al., 2020), Iran (Jahantigh et al., 2020), Malaysia (Ibrahim et al., 2021), and Egypt (Elmonir et al., 2021). Mechanistically, resistance to tetracycline has been associated with increased expression of the *mar* operon, where tetracycline-induced mutations enhance MarA-mediated transcriptional regulation, contributing to broad-spectrum multidrug resistance (Ruiz and Levy, 2010). In this context, the majority of the isolates demonstrated resistance to three or more antibiotics and were thus classified as multidrug-resistant (MDR) (Magiorakos et al., 2012). The emergence of AMR in duck can be attributed to several sources, including contaminated feed ingredients, indiscriminate antimicrobial use in adjacent poultry and livestock systems, and environmental exposures such as agricultural runoff and sewage contamination (Pruden et al., 2004; Manyi-Loh et al., 2018). Additionally, free-ranging ducks often share aquatic ecosystems with humans - where bathing, washing clothes, and other routine activities occur - creating potential hotspots for AMR dissemination and reinforcing the human-animal-environment interface (Huijbers et al., 2015).

In the present study, highly significant (*p* < 0.001) positive associations among biofilm formation, antibiotic resistance, and virulence gene (*invA*) prevalence was observed in *Salmonella* isolates. Earlier, such type of intriguing relationship between ESBL and *AmpC* production and biofilm formation was reported by Subramanian et al. (2012). The association study suggests that biofilm formation may serve as a predictive marker for resistance and virulence, making it a potential diagnostic feature for identifying high-risk *Salmonella* strains in avian sources. This integrated approach could facilitate more targeted interventions and surveillance strategies within duck health management frameworks. One-Sample Binomial test yielded a highly statistical significant result (*p* < 0.001), indicating that the observed proportion of virulent isolates (32%) is significantly lower than the expected prevalence, suggesting a lower-than-expected burden of virulent strains in the sampled population. This could imply a relatively moderate zoonotic threat from these isolates under current conditions.

The nucleotide sequencing of the PCR products revealed that the variants of the ß-lactamase circulating in the duck population were *bla*_*CTX*−*M*−15_, followed by *bla*_*SHV*−215_, *bla*_*TEM*−72_, *bla*_*CTX*−*M*−28_, *bla*_*CTX*−*M*−82_, *bla*_*SHV*−27_, *bla*_*SHV*−45_, *bla*_*SHV*−191_, *bla*_*SHV*−2_, *bla*_*SHV*−249_, *bla*_*SHV*−99_, *bla*_*TEM*−1_, and one *bla*_*TEM*−164_. A previous study reported TEM-1 as the most frequently detected β-lactamase among *Salmonella* strains isolated from poultry and poultry products in the Netherlands (Hasman et al., 2005). Although TEM-1 is not classified as a classical ESBL, it is frequently reported in human clinical isolates worldwide, and its encoded enzyme has occasionally exhibited extended-spectrum β-lactamase activity (Paterson and Bonomo, 2005; Carattoli et al., 2008). The presence of *bla*_*CTX*−*M*−15_ is predominantly associated with clinical *Enterobacteriaceae* isolates from both human and animal origins globally (Haenni et al., 2012). *Enterobacteriaceae* members harboring *bla*_*CTX*−*M*−15_ have previously been reported in poultry across various geographic regions (Blaak et al., 2015; Blanc et al., 2006; Kawamura et al., 2014).

The SHV cluster, in the dendrogram comprising 21 sequences, demonstrated substantial phylogenetic diversity across geographical regions and host species. The cluster begins with SHV-27_EU418911 (clinical, Australia) and extends to SHV-2_LC876679 (duck, India-WB) at the upper end. SHV-27 emerged as the most frequently detected variant (*n* = 7), identified in clinical isolates (Australia, Myanmar, Bangladesh, China), as well as poultry (LC653140, LC738864) and notably, a duck isolate from West Bengal (LC774700). This distribution underscores the widespread dissemination of SHV-27 across both human and avian reservoirs. Three SHV-2 sequences, including isolates from duck (LC876679_W.B.), clinical origin (MF402903_CHINA), and pig (EU376967_CHINA), reveal cross-species transmission potential, particularly between livestock and human-associated settings. Two SHV-215–positive isolates (LC774610 from duck and LC876924 from feed, both West Bengal) and two SHV-191–positive duck isolates (LC875420_W.B. and KP868754_CHINA) reflect the persistence of these variants in avian environments, with occasional clinical connections. Additionally, SHV-45 was detected in three isolates, including two *Escherichia* and *Klebsiella* strains from poultry and clinical sources, while a duck-origin SHV-45 isolate (LC876923_W.B.) was notably positioned within the TEM cluster. The misplacement of SHV-45 within the TEM cluster observed in our phylogeny may be attributed to historical recombination or domain sharing between SHV and TEM β-lactamases. Previous studies have documented such genetic exchange events, supporting the plausibility of interfamily phylogenetic overlaps (Barlow et al., 2009). Other SHV types, viz. SHV-249 (from duck and clinical isolates) and SHV-99 (from duck and clinical sources) also appeared in both human and animal contexts, reinforcing the zoonotic relevance of these β-lactamase variants. The phylogenetic interspersion of SHV sequences from wildlife, poultry, feed, and clinical settings suggest active gene flow and environmental circulation. The high diversity of SHV types, especially in duck isolates, emphasizes the role of avian reservoirs in the maintenance and evolution of SHV-type ESBLs, with potential spill-over into human populations.

The TEM gene cluster revealed in the phylogenetic analysis spans a diverse range of TEM variants, from TEM-1 to TEM-164, distributed across poultry, ducks, feed, bats, and clinical sources. The cluster begins with TEM-1_LC656923_W.B, isolated from poultry, and progresses through several *Salmonella* and *Escherichia* isolates of West Bengal origin, including those from duck (LC878794), feed (LC878796), and bat (LC877839). This suggests regional circulation and probable environmental exchange of TEM-type ESBL genes. The cluster also includes TEM-72 variants from diverse sources: *Salmonella* (LC878793) and *Klebsiella* (LC878026) in bats, and even *Morganella morganii* from a human clinical case in Italy (AF157553), highlighting the broad host range and interspecies movement of this allele. Toward the terminal end, the inclusion of TEM-164 isolates from bat (LC878795_W.B.), *E. coli* (LC878028_W.B.), and a clinical isolate from Saudi Arabia (MT928786) reflects the global dissemination and possible evolution of TEM variants toward higher resistance profiles. The tight clustering of these sequences across different hosts and ecological niches underscores the mobility and adaptability of TEM-type β-lactamases. Their detection in both wildlife and domestic environments signals the potential role of non-clinical reservoirs in sustaining and spreading antimicrobial resistance determinants.

The phylogenetic analysis revealed a distinct CTX-M cluster comprising 16 sequences, primarily dominated by CTX-M-15, CTX-M-28, and CTX-M-82 variants, across diverse hosts and sources. Notably, three CTX-M-82–positive isolates—EU545409 (clinical, China), GU477621 (dog, China), and LC875419 (duck, India-WB)—clustered closely, suggesting potential cross-species or cross-regional transmission. A tight grouping of CTX-M-15 isolates from ducks (LC774654, LC874701, LC874702) in West Bengal showed minimal divergence and were interspersed with clinical isolates from South Korea and Japan, including LC383367 from a giant panda, indicating possible international lineage sharing. Additionally, CTX-M-28 variants from duck, bat, and human clinical sources (LC874602, LC877130, EU531512) were phylogenetically linked, underscoring zoonotic potential. The presence of CTX-M-15 in *E. coli* from cow milk, poultry, dog, and cat in West Bengal further highlights horizontal gene transfer and the regional establishment of these alleles. Overall, the cluster reflects the widespread dissemination and ecological versatility of CTX-M genes, particularly CTX-M-15, across human, animal, and environmental reservoirs.

The analysis of outer membrane proteins (OMPs) from six distinct *Salmonella* spp. isolates revealed a diverse and variable banding pattern when subjected to SDS-PAGE analysis, with molecular weights ranging from 5 kDa to 109 kDa. Notably, the number of protein bands varied across the isolates, suggesting differences in their OMP compositions. The C-6d strain exhibited 15 distinct protein bands, while T-7b and T-18b strains displayed only 8 bands. T-10c and BF-2a strains exhibited 14 and 17 bands, respectively. The C-10a strain demonstrated the highest number of protein bands (21), indicating a potentially rich source of immunogenic proteins. Our findings align with previous studies identifying *Salmonella* spp. outer membrane proteins (OMPs) within the 18-43 kDa range (Singh et al., 2007), highlighting their importance in pathogenicity and vaccine development (Dehghani et al., 2013). Andrade et al. (1998) introduced the immunogenic OMP Omp-28 from *Salmonella* typhi, purified through chromatography. Arockiasamy and Krishnaswamy (2000) extracted OmpC from *S. typhi* Ty21a, confirming its purity via SDS-PAGE. Sarowska et al. (2010) found that ESBL-producing *Salmonella* transconjugants were highly sensitive to normal human serum (NHS), unlike their parental strains. SDS-PAGE analysis revealed distinct OMP patterns, underscoring the role of OMPs in serum resistance and their potential as diagnostic targets. These collective findings underscore the importance of OMP studies in understanding *Salmonella* pathogenicity and developing targeted interventions. The immunogenicity of the OMPs was assessed using ELISA, with the C-10a strain showing the highest antibody titre. This indicates that the OMPs from the C-10a strain are highly immunogenic. Previous studies have characterized the immunogenicity of *Salmonella* outer membrane proteins (OMPs) like porin C from *S. typhimurium* using ELISA (Prejit et al., 2013). Verdugo-Rodrigues et al. (1993) applied an iELISA to distinguish typhoid fever patients from healthy individuals based on antibody responses to *S. typhi* OMPs, with higher absorbance in patients (average 1.52) compared to healthy subjects (average 0.30). Monoclonal antibodies targeting *S. enteritidis* OMPs were tested against 57 serovars, detecting *Salmonella* in clinical samples with specificity (Kerr et al., 1992). Prakash et al. (2005) developed an iELISA to measure antibody responses in chickens infected with *S. gallinarum*, showing higher titres in infected flocks compared to vaccinated ones, mirrored in egg yolk antibody levels. Western blotting of six *Salmonella* spp. isolates identified immunogenic OMPs with molecular weights ranging from 20 to 85 kDa. Four isolates displayed multiple immunogenic OMPs, underscoring their potential as targets immunological studies. Notably, among the six selected OMPs, four showed significant immunoreactivity when exposed to hyperimmune serum. The OMPs T-18b and T-10c exhibited reactivity at 69 and 35 kDa, with T-10c also showing reactivity at 33 kDa. The C-10a OMP demonstrated the highest intensity of immunoreactivity across various bands (85, 75, 69, 35, 33, and 20 kDa). BF-2a OMP showed distinct reactivity at 69, 51, 44, 35, and 33 kDa. El-Fakar and Rabie (2009) investigated *Salmonella* infections in chickens, identifying shared antigen bands via SDS-electrophoresis, particularly between *S. typhimurium* and *S. enteritidis* in the 20-45 kDa range. Western blot analysis revealed *S. enteritidis*-specific bands at 17-31 kDa, similar to findings in this study. *S. pullorum* and *S. gallinarum* showed bands at 14.4 kDa and 24 kDa, consistent with present results, while *S. typhimurium* displayed a unique 24 kDa band. Seleim et al. (2004) observed a comparable band pattern in calves infected with *Salmonella* serovars, with shared antigen bands from 20 kDa to 45 kDa. Western blot analysis showed specific bands for each serovar, highlighting similar findings to this study and emphasizing the robustness of immunogenic patterns observed across different studies. These findings suggest that certain cross-reactive polypeptides (35 and 69 kDa) could serve as diagnostic markers for identifying *Salmonella* spp. with specific traits like antibiotic resistance, biofilm formation, and virulence gene expression. The DOT-ELISA assay was employed to qualitatively assess the presence of anti-*Salmonella* antibodies in serum samples from healthy rabbits and from rabbits experimentally inoculated with *Salmonella* spp. exhibiting antibiotic resistance (ESBL), virulence, and biofilm formation properties. The assay results demonstrated a distinct difference between the control and test groups. The successful application of DOT-ELISA in this study highlights its potential as a valuable tool for monitoring *Salmonella* spp. exhibiting antibiotic resistance (ESBL), virulence, and biofilm formation properties. Further studies are warranted to explore the application of DOT-ELISA using sandwich principle to capture 69 and 35 kDa OMP of *Salmonella* spp.

The present study has got certain limitations. It was restricted to duck-origin isolates, and therefore its direct relevance to the human sector remains limited. Detailed information regarding routine biosecurity practices and antibiotic usage at the farm level was not systematically collected during sampling and therefore was not included in the analysis. Biofilm-negative and virulence-negative isolates were not separately analyzed through ABST, which could have provided a baseline resistance profile. In addition, molecular typing approaches such as MLST and serotyping were not performed that impede our ability to comprehensively characterize transmission dynamics and elucidate the broader One Health interface.

## 5 Conclusion

The present investigation demonstrates for the first time that healthy ducks and their surrounding environments in one of the eastern Indian states, West Bengal, serve as reservoirs for *Salmonella* strains exhibiting a combined profile of antimicrobial resistance, biofilm formation, and virulence. Among 436 PCR-confirmed isolates, 42.20% were ESBL producers carrying *bla*_*TEM*_(36.47%), *bla*_*CTX*−*M*_(20.64%), *bla*_*SHV*_(17.66%), and *bla*_*AmpC*_(32.57%), with sequence analysis identifying multiple high-risk variants. Biofilm-associated genes (*csgA*: 54.59%; *sdiA*: 52.52%; *rpoS*: 80.28%; *rcsA*: 63.76%) and the *invA* virulence gene (32.34%) were widely detected, while multi-drug resistance was common, marked by high resistance to tetracycline (84.62%) and cefixime (80.77%). Phylogenetic analysis suggested interspecies transmission potential, clustering ESBL gene variants from avian, animal, and clinical sources. SDS-PAGE and Western blotting revealed two immunodominant unique polypeptides (69 and 35 kDa) in representative MDR, biofilm-forming, virulent isolates, highlighting their potential as diagnostic markers for surveillance of high-risk avian *Salmonella*.

## Data Availability

The original contributions presented in the study are included in the article/supplementary material, further inquiries can be directed to the corresponding authors.
